# *N*-(3-Bromo-1,4-dioxo-1,4-dihydro-2-naphth­yl)-4-fluoro-*N*-(4-fluoro­benzo­yl)benzamide

**DOI:** 10.1107/S1600536809000117

**Published:** 2009-01-10

**Authors:** Emmanuel S. Akinboye, Ray J. Butcher, Dwayne A. Wright, Yakini Brandy, Oladapo Bakare

**Affiliations:** a Department of Chemistry, Howard University, 525 College Street NW, Washington, DC 20059, USA

## Abstract

In the title compound, C_24_H_12_BrF_2_NO_4_, synthesized from 2-amino-3-bromo-1,4-naphthoquinone and 4-fluoro­benzoyl chloride, the two *p*-fluoro­phenyl rings are inclined at 73.9 (1) and 73.6 (1)° to the naphthoquinone ring system. The two imido carbonyl O atoms are *anti* to each other, while the fluoro­phenyl rings are located opposite each other, connected to the imide group in a funnel-like arrangement. This conformation allows the fluorine groups be oriented slightly away from each other. An examination of the packing shows a close inter­molecular F⋯O contact of 2.982 (5) Å and a Br⋯O contact of 2.977 (4) Å. In addition, the mol­ecules are linked by weak inter­molecular C—H⋯O and C—H⋯F inter­actions.

## Related literature

For similar structures, see: Lien *et al.* (1997 ▶); Huang *et al.* (2005 ▶); Bakare *et al.* (2003 ▶); Akinboye *et al.* (2009 ▶); Win *et al.* (2005 ▶); Rubin-Preminger *et al.* (2004 ▶). For general background, see: Berhe *et al.* (2008 ▶).
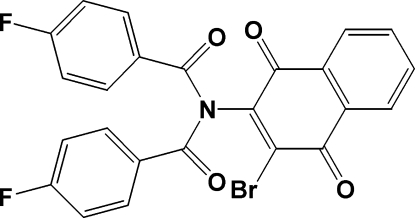


## Experimental

### 

#### Crystal data

C_24_H_12_BrF_2_NO_4_
*M* *_r_* = 496.26
Monoclinic, 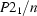

*a* = 14.5931 (3) Å
*b* = 6.6471 (1) Å
*c* = 20.6324 (4) Å
β = 98.407 (2)°
*V* = 1979.88 (6) Å^3^
*Z* = 4
Cu *K*α radiation
μ = 3.30 mm^−1^
*T* = 200 (2) K
0.53 × 0.48 × 0.32 mm

#### Data collection

Oxford Diffraction Gemini R diffractometer
Absorption correction: multi-scan (*CrysAlis RED*; Oxford Diffraction, 2007 ▶) *T*_min_ = 0.230, *T*_max_ = 0.348
7835 measured reflections
3802 independent reflections
3362 reflections with *I* > 2σ(*I*)
*R*_int_ = 0.023

#### Refinement

*R*[*F* ^2^ > 2σ(*F* ^2^)] = 0.056
*wR*(*F* ^2^) = 0.158
*S* = 1.14
3802 reflections
289 parameters
H-atom parameters constrained
Δρ_max_ = 1.29 e Å^−3^
Δρ_min_ = −0.51 e Å^−3^

### 

Data collection: *CrysAlisPro* (Oxford Diffraction, 2007 ▶); cell refinement: *CrysAlisPro*; data reduction: *CrysAlisPro*; program(s) used to solve structure: *SHELXS97* (Sheldrick, 2008 ▶); program(s) used to refine structure: *SHELXL97* (Sheldrick, 2008 ▶); molecular graphics: *SHELXTL* (Sheldrick, 2008 ▶); software used to prepare material for publication: *SHELXTL*.

## Supplementary Material

Crystal structure: contains datablocks global, I. DOI: 10.1107/S1600536809000117/bq2117sup1.cif

Structure factors: contains datablocks I. DOI: 10.1107/S1600536809000117/bq2117Isup2.hkl

Additional supplementary materials:  crystallographic information; 3D view; checkCIF report

## Figures and Tables

**Table 1:** Hydrogen-bond geometry (Å, °)

*D*—H⋯*A*	*D*—H	H⋯*A*	*D*⋯*A*	*D*—H⋯*A*
C5—H5*A*⋯O1*B*^i^	0.95	2.56	3.297 (6)	135
C4—H4*A*⋯F1*A*^ii^	0.95	2.40	3.266 (6)	151

Symmetry codes: (i) 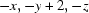; (ii) 
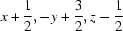
.

**Fig. 1.**
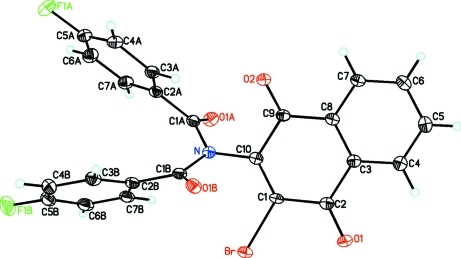
View of (I) showing the atom-labelling scheme. Displacement ellipsoids are drawn at the 20% probability level.

**Fig. 2.**
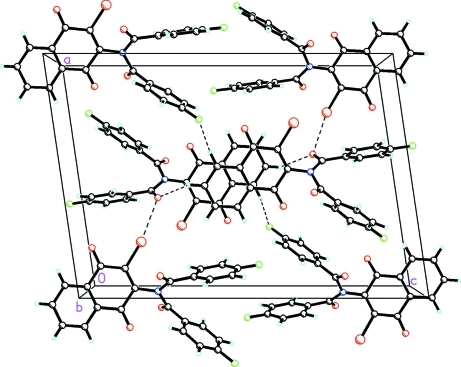
View of the packing viewed down the a axis. Dashed bonds show weak C—H···F interactions as well as the close Br···O intermolecular contact.

## supplementary crystallographic information

### Comment

We have developed some imido-substituted 2-chloro-1,4-naphthoquinones with cytotoxic activities on some cancer cell lines (Bakare *et al.*, 2003; Berhe *et al.*, 2008); and have recently reported on the crystal structure of *N*-(3-bromo-1,4-dioxo- 1,4-dihydro-naphthalen-2-yl)-2-chloro-*N*-(2-chloro-benzoyl)-benzamide (Akinboye *et al.*, 2009). In continuation of our work, the title compound C_24_H_12_BrF_2_NO_4_, (I), was synthesized as a potential anticancer agent.

The crystal structure shows that the two *p*-fluorophenyl rings are inclined at 73.9 (1) and 73.6 (1)° to the naphthoquinone ring. The two imido carbonyl oxygen atoms are anti-to each other, while fluorophenyl rings are placed facing each other and connected to the imide moiety in a funnel-like arrangement. This conformation allowed the fluorine groups in the *para* position of each fluorophenyl ring to be oriented slightly away from each other. An examination of the packing shows a close contact between F1A and O2 at (1/2 - *x*, 1/2 + *y*, 1/2 - *z*) (2.982 (5)Å) and between C2 and O1B at (1/2 - *x*, -1/2 + *y*, 1/2 - *z*) (2.977 (4)Å). In addition, the molecules are linked by weak intermolecular C—H···O and C—H···F interactions (Table 1).

### Experimental

To a solution 2-amino-3-bromo-1,4-naphthoquinone (300 mg, 1.19 mmol) in dry THF was added NaH (68.64 mg 2.86 mmol) and the mixture was stirred for 15 minutes. 4-Fluoro-benzoylchloride (0.35 ml, 2.86 mmol) was added thereafter and this mixture was stirred at room temperature for 16–24 hr under argon. The solvent was removed *in vacuo* and the solid residue was dissolved in dichloromethane (40 ml). The resultant solution was washed with water (3 *x* 15 ml), saturated NaCl solution (2 *x* 15 ml) and dried over anhydrous magnesium sulfate. The solvent was removed *in vacuo* and the residue recrystallized from ethyl acetate to obtain a yellow solid (391.0 g m). Further recrystallization was carried out in ethanol to furnish the title imide (340.2 mg, 57%).

### Refinement

The methyl H atoms were constrained to an ideal geometry with C—H distances of 0.98 Å and *U*_iso_(H) = 1.5*U*_eq_(C), but each group was allowed to rotate freely about its C—C bond. All other H atoms were placed in geometrically idealized positions and constrained to ride on their parent atoms with C—H distances in the range 0.95–1.00 Å and *U*_iso_(H) = 1.2*U*_eq_(C).

### Crystal data

**Table:**

C_24_H_12_BrF_2_NO_4_	*F*(000) = 992
*M**_r_* = 496.26	*D*_x_ = 1.665 Mg m^−^^3^
Monoclinic, *P*2_1_/*n*	Cu *K*α radiation, λ = 1.54184 Å
Hall symbol: -P 2yn	Cell parameters from 5844 reflections
*a* = 14.5931 (3) Å	θ = 4.0–73.4°
*b* = 6.6471 (1) Å	µ = 3.30 mm^−^^1^
*c* = 20.6324 (4) Å	*T* = 200 K
β = 98.407 (2)°	Prism, pale yellow
*V* = 1979.88 (6) Å^3^	0.53 × 0.48 × 0.32 mm
*Z* = 4	

### Data collection

**Table:**

Oxford Diffraction Gemini R diffractometer	3802 independent reflections
Radiation source: fine-focus sealed tube	3362 reflections with *I* > 2σ(*I*)
graphite	*R*_int_ = 0.023
Detector resolution: 10.5081 pixels mm^-1^	θ_max_ = 73.6°, θ_min_ = 4.0°
φ and ω scans	*h* = −17→18
Absorption correction: multi-scan (*CrysAlis RED*; Oxford Diffraction, 2007)	*k* = −8→5
*T*_min_ = 0.230, *T*_max_ = 0.348	*l* = −25→25
7835 measured reflections	

### Refinement

**Table:**

Refinement on *F*^2^	Primary atom site location: structure-invariant direct methods
Least-squares matrix: full	Secondary atom site location: difference Fourier map
*R*[*F*^2^ > 2σ(*F*^2^)] = 0.056	Hydrogen site location: inferred from neighbouring sites
*wR*(*F*^2^) = 0.158	H-atom parameters constrained
*S* = 1.14	*w* = 1/[σ^2^(*F*_o_^2^) + (0.0639*P*)^2^ + 6.5199*P*] where *P* = (*F*_o_^2^ + 2*F*_c_^2^)/3
3802 reflections	(Δ/σ)_max_ < 0.001
289 parameters	Δρ_max_ = 1.29 e Å^−^^3^
0 restraints	Δρ_min_ = −0.51 e Å^−^^3^

### Special details

**Table:**

Geometry. All e.s.d.'s (except the e.s.d. in the dihedral angle between two l.s. planes) are estimated using the full covariance matrix. The cell e.s.d.'s are taken into account individually in the estimation of e.s.d.'s in distances, angles and torsion angles; correlations between e.s.d.'s in cell parameters are only used when they are defined by crystal symmetry. An approximate (isotropic) treatment of cell e.s.d.'s is used for estimating e.s.d.'s involving l.s. planes.
Refinement. Refinement of *F*^2^ against ALL reflections. The weighted *R*-factor *wR* and goodness of fit *S* are based on *F*^2^, conventional *R*-factors *R* are based on *F*, with *F* set to zero for negative *F*^2^. The threshold expression of *F*^2^ > σ(*F*^2^) is used only for calculating *R*-factors(gt) *etc*. and is not relevant to the choice of reflections for refinement. *R*-factors based on *F*^2^ are statistically about twice as large as those based on *F*, and *R*- factors based on ALL data will be even larger.

### Fractional atomic coordinates and isotropic or equivalent isotropic displacement parameters (Å^2^)

**Table:**

	*x*	*y*	*z*	*U*_iso_*/*U*_eq_	
Br	0.22095 (3)	0.70099 (8)	0.19146 (2)	0.0444 (2)	
F1A	−0.2690 (2)	0.9709 (5)	0.41727 (16)	0.0599 (8)	
F1B	0.1251 (3)	0.7110 (6)	0.52944 (16)	0.0752 (11)	
O1	0.1976 (2)	0.7543 (6)	0.04619 (17)	0.0448 (8)	
O2	−0.1290 (2)	0.8464 (5)	0.12723 (16)	0.0439 (8)	
O1A	−0.0683 (2)	0.4826 (5)	0.21656 (17)	0.0479 (8)	
O1B	0.0933 (2)	1.0688 (5)	0.24341 (15)	0.0424 (7)	
N	0.0163 (2)	0.7707 (6)	0.22161 (18)	0.0358 (8)	
C1	0.1109 (3)	0.7445 (7)	0.1341 (2)	0.0330 (9)	
C2	0.1213 (3)	0.7515 (6)	0.0634 (2)	0.0335 (9)	
C3	0.0344 (3)	0.7515 (6)	0.0152 (2)	0.0336 (9)	
C4	0.0391 (3)	0.7337 (7)	−0.0509 (2)	0.0386 (10)	
H4A	0.0976	0.7218	−0.0656	0.046*	
C5	−0.0414 (4)	0.7333 (6)	−0.0961 (2)	0.0404 (10)	
H5A	−0.0380	0.7208	−0.1415	0.048*	
C6	−0.1272 (3)	0.7512 (7)	−0.0744 (2)	0.0395 (10)	
H6A	−0.1824	0.7503	−0.1051	0.047*	
C7	−0.1317 (3)	0.7702 (7)	−0.0086 (2)	0.0375 (10)	
H7A	−0.1903	0.7830	0.0059	0.045*	
C8	−0.0513 (3)	0.7706 (6)	0.0370 (2)	0.0333 (9)	
C9	−0.0579 (3)	0.7970 (6)	0.1070 (2)	0.0343 (9)	
C10	0.0287 (3)	0.7658 (6)	0.1550 (2)	0.0324 (9)	
C1A	−0.0557 (3)	0.6469 (7)	0.2405 (2)	0.0372 (10)	
C2A	−0.1127 (3)	0.7365 (7)	0.2871 (2)	0.0363 (9)	
C3A	−0.1389 (3)	0.9372 (7)	0.2822 (2)	0.0389 (10)	
H3AA	−0.1194	1.0199	0.2493	0.047*	
C4A	−0.1936 (3)	1.0168 (8)	0.3254 (2)	0.0424 (10)	
H4AA	−0.2131	1.1532	0.3222	0.051*	
C5A	−0.2187 (3)	0.8922 (9)	0.3732 (2)	0.0457 (11)	
C6A	−0.1944 (4)	0.6934 (8)	0.3788 (3)	0.0492 (12)	
H6AA	−0.2135	0.6120	0.4122	0.059*	
C7A	−0.1415 (3)	0.6137 (8)	0.3348 (2)	0.0444 (11)	
H7AA	−0.1248	0.4754	0.3371	0.053*	
C1B	0.0646 (3)	0.9152 (7)	0.2643 (2)	0.0354 (9)	
C2B	0.0816 (3)	0.8578 (8)	0.3349 (2)	0.0382 (10)	
C3B	0.0772 (3)	1.0046 (8)	0.3817 (2)	0.0452 (11)	
H3BA	0.0641	1.1397	0.3685	0.054*	
C4B	0.0918 (4)	0.9575 (10)	0.4476 (3)	0.0529 (13)	
H4BA	0.0880	1.0576	0.4799	0.063*	
C5B	0.1121 (4)	0.7597 (10)	0.4649 (3)	0.0544 (14)	
C6B	0.1210 (3)	0.6130 (9)	0.4200 (3)	0.0517 (13)	
H6BA	0.1377	0.4798	0.4337	0.062*	
C7B	0.1051 (3)	0.6619 (8)	0.3541 (2)	0.0430 (11)	
H7BA	0.1103	0.5616	0.3220	0.052*	

### Atomic displacement parameters (Å^2^)

**Table:**

	*U*^11^	*U*^22^	*U*^33^	*U*^12^	*U*^13^	*U*^23^
Br	0.0270 (3)	0.0572 (4)	0.0469 (3)	0.0056 (2)	−0.00134 (19)	0.0002 (2)
F1A	0.0519 (17)	0.068 (2)	0.0664 (19)	0.0020 (15)	0.0300 (15)	−0.0024 (16)
F1B	0.084 (3)	0.098 (3)	0.0405 (17)	−0.006 (2)	−0.0033 (16)	0.0140 (17)
O1	0.0291 (15)	0.057 (2)	0.0496 (19)	0.0045 (14)	0.0103 (13)	−0.0014 (16)
O2	0.0304 (15)	0.058 (2)	0.0429 (17)	0.0055 (15)	0.0043 (13)	−0.0034 (15)
O1A	0.0466 (19)	0.0395 (19)	0.059 (2)	−0.0065 (15)	0.0130 (16)	−0.0070 (16)
O1B	0.0404 (17)	0.0418 (18)	0.0429 (17)	−0.0065 (14)	−0.0012 (13)	−0.0001 (14)
N	0.0286 (17)	0.042 (2)	0.0366 (19)	−0.0023 (15)	0.0036 (14)	−0.0002 (15)
C1	0.0211 (18)	0.033 (2)	0.043 (2)	0.0010 (16)	−0.0030 (16)	−0.0005 (17)
C2	0.032 (2)	0.025 (2)	0.043 (2)	0.0023 (16)	0.0040 (17)	−0.0023 (17)
C3	0.033 (2)	0.026 (2)	0.041 (2)	0.0018 (17)	0.0048 (17)	0.0015 (17)
C4	0.044 (3)	0.029 (2)	0.043 (2)	0.0020 (19)	0.0075 (19)	0.0007 (18)
C5	0.056 (3)	0.023 (2)	0.041 (2)	0.0026 (19)	0.006 (2)	−0.0002 (17)
C6	0.045 (3)	0.027 (2)	0.043 (2)	−0.0013 (19)	−0.006 (2)	0.0010 (18)
C7	0.031 (2)	0.034 (2)	0.046 (2)	0.0018 (18)	0.0000 (18)	0.0016 (18)
C8	0.030 (2)	0.027 (2)	0.042 (2)	−0.0008 (16)	0.0012 (17)	0.0009 (17)
C9	0.027 (2)	0.033 (2)	0.042 (2)	−0.0026 (17)	0.0021 (17)	0.0000 (17)
C10	0.031 (2)	0.026 (2)	0.040 (2)	−0.0008 (16)	0.0012 (17)	0.0000 (16)
C1A	0.030 (2)	0.042 (3)	0.039 (2)	−0.0005 (19)	0.0017 (17)	0.0016 (19)
C2A	0.027 (2)	0.042 (2)	0.039 (2)	−0.0057 (18)	0.0015 (17)	0.0000 (18)
C3A	0.030 (2)	0.046 (3)	0.040 (2)	−0.0022 (19)	0.0029 (17)	0.0060 (19)
C4A	0.030 (2)	0.046 (3)	0.051 (3)	0.0027 (19)	0.0042 (19)	0.003 (2)
C5A	0.030 (2)	0.062 (3)	0.046 (3)	−0.003 (2)	0.0092 (19)	−0.002 (2)
C6A	0.045 (3)	0.051 (3)	0.055 (3)	−0.005 (2)	0.015 (2)	0.009 (2)
C7A	0.039 (2)	0.041 (3)	0.054 (3)	−0.004 (2)	0.009 (2)	0.004 (2)
C1B	0.0225 (18)	0.042 (3)	0.041 (2)	−0.0030 (17)	0.0015 (16)	−0.0013 (19)
C2B	0.0250 (19)	0.049 (3)	0.040 (2)	−0.0066 (19)	0.0008 (16)	0.000 (2)
C3B	0.036 (2)	0.050 (3)	0.049 (3)	−0.004 (2)	0.003 (2)	−0.002 (2)
C4B	0.046 (3)	0.069 (4)	0.043 (3)	−0.008 (3)	0.004 (2)	−0.007 (2)
C5B	0.040 (3)	0.083 (4)	0.039 (3)	−0.012 (3)	−0.001 (2)	0.010 (3)
C6B	0.035 (2)	0.062 (3)	0.056 (3)	0.002 (2)	−0.002 (2)	0.011 (3)
C7B	0.029 (2)	0.051 (3)	0.048 (3)	0.003 (2)	−0.0004 (18)	0.002 (2)

### Geometric parameters (Å, °)

**Table:**

Br—C1	1.873 (4)	C9—C10	1.503 (6)
F1A—C5A	1.353 (6)	C1A—C2A	1.486 (6)
F1B—C5B	1.356 (6)	C2A—C3A	1.387 (7)
O1—C2	1.217 (5)	C2A—C7A	1.390 (7)
O2—C9	1.218 (5)	C3A—C4A	1.385 (7)
O1A—C1A	1.201 (6)	C3A—H3AA	0.9500
O1B—C1B	1.207 (6)	C4A—C5A	1.378 (7)
N—C10	1.412 (6)	C4A—H4AA	0.9500
N—C1B	1.419 (6)	C5A—C6A	1.369 (8)
N—C1A	1.433 (6)	C6A—C7A	1.381 (7)
C1—C10	1.340 (6)	C6A—H6AA	0.9500
C1—C2	1.489 (6)	C7A—H7AA	0.9500
C2—C3	1.493 (6)	C1B—C2B	1.492 (6)
C3—C4	1.382 (6)	C2B—C3B	1.381 (7)
C3—C8	1.395 (6)	C2B—C7B	1.390 (7)
C4—C5	1.388 (7)	C3B—C4B	1.380 (7)
C4—H4A	0.9500	C3B—H3BA	0.9500
C5—C6	1.395 (7)	C4B—C5B	1.383 (9)
C5—H5A	0.9500	C4B—H4BA	0.9500
C6—C7	1.374 (7)	C5B—C6B	1.365 (9)
C6—H6A	0.9500	C6B—C7B	1.384 (7)
C7—C8	1.393 (6)	C6B—H6BA	0.9500
C7—H7A	0.9500	C7B—H7BA	0.9500
C8—C9	1.473 (6)		
			
C10—N—C1B	119.8 (4)	C7A—C2A—C1A	118.6 (4)
C10—N—C1A	117.0 (4)	C4A—C3A—C2A	119.9 (4)
C1B—N—C1A	122.5 (4)	C4A—C3A—H3AA	120.0
C10—C1—C2	122.5 (4)	C2A—C3A—H3AA	120.0
C10—C1—Br	122.5 (3)	C5A—C4A—C3A	118.0 (5)
C2—C1—Br	115.0 (3)	C5A—C4A—H4AA	121.0
O1—C2—C1	121.0 (4)	C3A—C4A—H4AA	121.0
O1—C2—C3	122.0 (4)	F1A—C5A—C6A	118.4 (5)
C1—C2—C3	117.0 (4)	F1A—C5A—C4A	118.4 (5)
C4—C3—C8	120.1 (4)	C6A—C5A—C4A	123.2 (5)
C4—C3—C2	119.9 (4)	C5A—C6A—C7A	118.6 (5)
C8—C3—C2	120.0 (4)	C5A—C6A—H6AA	120.7
C3—C4—C5	120.3 (4)	C7A—C6A—H6AA	120.7
C3—C4—H4A	119.8	C6A—C7A—C2A	119.7 (5)
C5—C4—H4A	119.8	C6A—C7A—H7AA	120.1
C4—C5—C6	119.7 (4)	C2A—C7A—H7AA	120.1
C4—C5—H5A	120.2	O1B—C1B—N	121.2 (4)
C6—C5—H5A	120.2	O1B—C1B—C2B	123.3 (4)
C7—C6—C5	120.0 (4)	N—C1B—C2B	115.4 (4)
C7—C6—H6A	120.0	C3B—C2B—C7B	119.9 (4)
C5—C6—H6A	120.0	C3B—C2B—C1B	119.0 (4)
C6—C7—C8	120.7 (4)	C7B—C2B—C1B	121.1 (4)
C6—C7—H7A	119.7	C4B—C3B—C2B	120.8 (5)
C8—C7—H7A	119.7	C4B—C3B—H3BA	119.6
C7—C8—C3	119.3 (4)	C2B—C3B—H3BA	119.6
C7—C8—C9	119.6 (4)	C3B—C4B—C5B	117.8 (5)
C3—C8—C9	121.1 (4)	C3B—C4B—H4BA	121.1
O2—C9—C8	123.4 (4)	C5B—C4B—H4BA	121.1
O2—C9—C10	119.2 (4)	F1B—C5B—C6B	118.9 (6)
C8—C9—C10	117.4 (4)	F1B—C5B—C4B	118.3 (5)
C1—C10—N	124.3 (4)	C6B—C5B—C4B	122.8 (5)
C1—C10—C9	120.6 (4)	C5B—C6B—C7B	118.7 (5)
N—C10—C9	115.0 (4)	C5B—C6B—H6BA	120.6
O1A—C1A—N	119.0 (4)	C7B—C6B—H6BA	120.6
O1A—C1A—C2A	124.3 (4)	C6B—C7B—C2B	119.9 (5)
N—C1A—C2A	116.6 (4)	C6B—C7B—H7BA	120.1
C3A—C2A—C7A	120.6 (4)	C2B—C7B—H7BA	120.1
C3A—C2A—C1A	120.8 (4)		
			
C10—C1—C2—O1	171.7 (4)	C1B—N—C1A—O1A	−150.5 (4)
Br—C1—C2—O1	−8.9 (6)	C10—N—C1A—C2A	−138.8 (4)
C10—C1—C2—C3	−9.3 (6)	C1B—N—C1A—C2A	31.9 (6)
Br—C1—C2—C3	170.1 (3)	O1A—C1A—C2A—C3A	−138.5 (5)
O1—C2—C3—C4	6.7 (7)	N—C1A—C2A—C3A	39.0 (6)
C1—C2—C3—C4	−172.3 (4)	O1A—C1A—C2A—C7A	39.8 (7)
O1—C2—C3—C8	−172.9 (4)	N—C1A—C2A—C7A	−142.8 (4)
C1—C2—C3—C8	8.1 (6)	C7A—C2A—C3A—C4A	0.5 (7)
C8—C3—C4—C5	−0.5 (6)	C1A—C2A—C3A—C4A	178.8 (4)
C2—C3—C4—C5	179.9 (4)	C2A—C3A—C4A—C5A	1.2 (7)
C3—C4—C5—C6	0.1 (7)	C3A—C4A—C5A—F1A	177.4 (4)
C4—C5—C6—C7	0.3 (7)	C3A—C4A—C5A—C6A	−1.8 (7)
C5—C6—C7—C8	−0.3 (7)	F1A—C5A—C6A—C7A	−178.6 (5)
C6—C7—C8—C3	−0.1 (7)	C4A—C5A—C6A—C7A	0.5 (8)
C6—C7—C8—C9	178.3 (4)	C5A—C6A—C7A—C2A	1.3 (8)
C4—C3—C8—C7	0.5 (6)	C3A—C2A—C7A—C6A	−1.8 (7)
C2—C3—C8—C7	−179.9 (4)	C1A—C2A—C7A—C6A	179.9 (4)
C4—C3—C8—C9	−177.8 (4)	C10—N—C1B—O1B	22.6 (6)
C2—C3—C8—C9	1.8 (6)	C1A—N—C1B—O1B	−147.9 (4)
C7—C8—C9—O2	−10.5 (7)	C10—N—C1B—C2B	−154.1 (4)
C3—C8—C9—O2	167.7 (4)	C1A—N—C1B—C2B	35.4 (6)
C7—C8—C9—C10	171.1 (4)	O1B—C1B—C2B—C3B	40.0 (6)
C3—C8—C9—C10	−10.6 (6)	N—C1B—C2B—C3B	−143.5 (4)
C2—C1—C10—N	−176.5 (4)	O1B—C1B—C2B—C7B	−137.4 (5)
Br—C1—C10—N	4.1 (6)	N—C1B—C2B—C7B	39.1 (6)
C2—C1—C10—C9	0.4 (6)	C7B—C2B—C3B—C4B	−3.1 (7)
Br—C1—C10—C9	−179.0 (3)	C1B—C2B—C3B—C4B	179.5 (4)
C1B—N—C10—C1	56.5 (6)	C2B—C3B—C4B—C5B	0.9 (7)
C1A—N—C10—C1	−132.5 (5)	C3B—C4B—C5B—F1B	−179.0 (5)
C1B—N—C10—C9	−120.6 (4)	C3B—C4B—C5B—C6B	2.1 (8)
C1A—N—C10—C9	50.5 (5)	F1B—C5B—C6B—C7B	178.2 (5)
O2—C9—C10—C1	−168.8 (4)	C4B—C5B—C6B—C7B	−2.9 (8)
C8—C9—C10—C1	9.6 (6)	C5B—C6B—C7B—C2B	0.7 (7)
O2—C9—C10—N	8.3 (6)	C3B—C2B—C7B—C6B	2.2 (7)
C8—C9—C10—N	−173.2 (4)	C1B—C2B—C7B—C6B	179.6 (4)
C10—N—C1A—O1A	38.7 (6)		

### Hydrogen-bond geometry (Å, °)

**Table:**

*D*—H···*A*	*D*—H	H···*A*	*D*···*A*	*D*—H···*A*
C5—H5A···O1B^i^	0.95	2.56	3.297 (6)	135
C4—H4A···F1A^ii^	0.95	2.40	3.266 (6)	151

Symmetry codes: (i) −*x*, −*y*+2, −*z*; (ii) *x*+1/2, −*y*+3/2, *z*−1/2.
